# Underlying cancer risk among patients with fatigue and other vague symptoms: a population-based cohort study in primary care

**DOI:** 10.3399/BJGP.2022.0371

**Published:** 2023-01

**Authors:** Becky White, Cristina Renzi, Matthew Barclay, Georgios Lyratzopoulos

**Affiliations:** Epidemiology of Cancer Healthcare and Outcomes (ECHO) Research Group, Department of Behavioural Science and Health, University College London, UK.; Epidemiology of Cancer Healthcare and Outcomes (ECHO) Research Group, Department of Behavioural Science and Health, University College London, UK, and associate professor, Faculty of Medicine, University Vita-Salute San Raffaele, Milan, Italy.; Epidemiology of Cancer Healthcare and Outcomes (ECHO) Research Group, Department of Behavioural Science and Health, University College London, UK.; Epidemiology of Cancer Healthcare and Outcomes (ECHO) Research Group, Department of Behavioural Science and Health, University College London, UK.

**Keywords:** early detection of cancer, fatigue, primary health care, signs and symptoms

## Abstract

**Background:**

Presenting to primary care with fatigue is associated with slightly increased cancer risk, although it is unknown how this varies in the presence of other ‘vague’ symptoms.

**Aim:**

To quantify cancer risk in patients with fatigue who present with other ‘vague’ symptoms in the absence of ‘alarm’ symptoms for cancer.

**Design and setting:**

Cohort study of patients presenting in UK primary care with new-onset fatigue during 2007–2015, using Clinical Practice Research Datalink data linked to national cancer registration data.

**Method:**

Patients presenting with fatigue without co-occurring alarm symptoms or anaemia were identified, who were further characterised as having co-occurrence of 19 other ‘vague’ potential cancer symptoms. Sex- and age-specific 9-month cancer risk for each fatigue–vague symptom cohort were calculated.

**Results:**

Of 285 382 patients presenting with new-onset fatigue, 84% (*n* = 239 846) did not have co-occurring alarm symptoms or anaemia. Of these, 38% (*n* = 90 828) presented with ≥1 of 19 vague symptoms for cancer. Cancer risk exceeded 3% in older males with fatigue combined with any of the vague symptoms studied. The age at which risk exceeded 3% was 59 years for fatigue–weight loss, 65 years for fatigue–abdominal pain, 67 years for fatigue–constipation, and 67 years for fatigue–other upper gastrointestinal symptoms. For females, risk exceeded 3% only in older patients with fatigue–weight loss (from 65 years), fatigue–abdominal pain (from 79 years), or fatigue–abdominal bloating (from 80 years).

**Conclusion:**

In the absence of alarm symptoms or anaemia, fatigue combined with specific vague presenting symptoms, alongside patient age and sex, can guide clinical decisions about referral for suspected cancer.

## INTRODUCTION

Many patients with cancer are diagnosed after presenting with vague symptoms,^1^ such as fatigue, which are characterised by lack of organ specificity and low positive predictive value for any single cancer type. Vague symptoms are not generally supported by urgent referral recommendations for suspected cancer under UK National Institute for Health and Care Excellence (NICE) guidelines, except for some specific patient groups and cancer sites. Patients diagnosed with cancer following presentation with these symptoms typically experience prolonged diagnostic intervals.^2^

Fatigue is a relatively common presenting symptom in primary care, being the primary complaint in an estimated 5–7% of consultations,^3^^–^6 and more commonly reported by females than males.^3^^,^^6^^,^^7^ It presents a diagnostic challenge, particularly regarding assessing the risk of underlying cancer.^4^^,^^5^^,^^8^^–^10 Although fatigue is reported by patients before diagnosis for a number of cancer sites,^11^^–^18 its predictive value for any single cancer site is low.^19^ Fatigue could also signal many other conditions, including self-limiting illnesses (for example, short-term post-viral fatigue), depression, chronic fatigue syndrome, autoimmune disease (for example, lupus), chronic infection (for example, hepatitis C), or a range of other causes (for example, hypothyroidism, vitamin deficiency, iron deficiency, coeliac disease).^4^^,^^8^^–^10^,^^20^

When new-onset fatigue accompanies an ‘alarm’ symptom for cancer, diagnostic management is typically straightforward. For example, in England, patients with ‘alarm’ symptoms for cancer can be referred to appropriate hospital specialties for urgent (‘two-week-wait’) investigation for suspected cancer (as per guidelines published by NICE).^11^^,^^21^ However, when patients with new-onset fatigue present with vague symptoms only, diagnostic management is less clear. GPs must discern which of these patients should nevertheless be investigated for cancer because of elevated risk associated with their demographic group or other vague signs and symptoms combined with fatigue, and whether to refer on to an urgent (‘two-week-wait’) pathway for suspected cancer or to a multidisciplinary diagnostic centre (‘rapid diagnostic centres’ in England).

More detailed evidence is needed to support such decision making. In a previous study, the authors of the current study quantified the risk of cancer diagnosis shortly after new-onset fatigue.^22^ How often fatigue presents alongside other symptoms and the associated risk of underlying cancer, however, is not known, although similar studies have been conducted in cohorts of patients with other vague symptoms, including weight loss or abdominal symptoms.^23^^–^25 Current evidence assessing cancer risk in patients with fatigue in combination with other presenting features is limited to specific cancer sites^12^^–^14^,^^17^^,^^19^^,^^26^ or symptom combinations.^23^^,^^27^ Furthermore, a detailed examination of cancer risk in patients presenting with new-onset fatigue in the absence of alarm symptoms would support GPs to identify which patients to refer in a group of patients for whom diagnostic management is particularly challenging.

**Table:** How this fits in

When patients present to GPs with new- onset fatigue and no alarm symptoms for cancer, optimal management is often unclear, as it is not known which of these patients may be at risk of having present but currently undetected cancer. The current study found that, among people who presented with fatigue but without alarm symptoms, the chance of underlying cancer exceeded risk referral thresholds of 3% in older males with fatigue combined with any of another 19 vague symptoms for cancer, and in older females with fatigue–weight loss, fatigue–abdominal pain, or fatigue-abdominal bloating. These findings can support diagnostic management and referral decisions for patients presenting with fatigue in the absence of alarm symptoms for cancer.

The aim of this study was therefore to estimate the short-term risk of incident diagnosis of any malignant neoplasm (excluding non-melanoma skin cancer) in patients who present with new-onset fatigue without accompanying alarm symptoms for cancer, according to combinations of other presenting vague symptoms.

## METHOD

### Study design and data source

A cohort study of patients with a record of fatigue presentation in primary care in England between January 2007 and April 2015 was conducted using electronic health records from the Clinical Practice Research Datalink (CPRD) GOLD (March 2019 database build). Data include patients’ recorded symptoms and sociodemographic information (age, sex). Cancers diagnosed during 2006–2015 were identified through linkage with cancer registration data held by the National Cancer Registration & Analysis Service (NCRAS) using an eight-step deterministic linkage algorithm including NHS number, sex, date of birth, and postcode.

### Symptom identification

**Figure 1.**
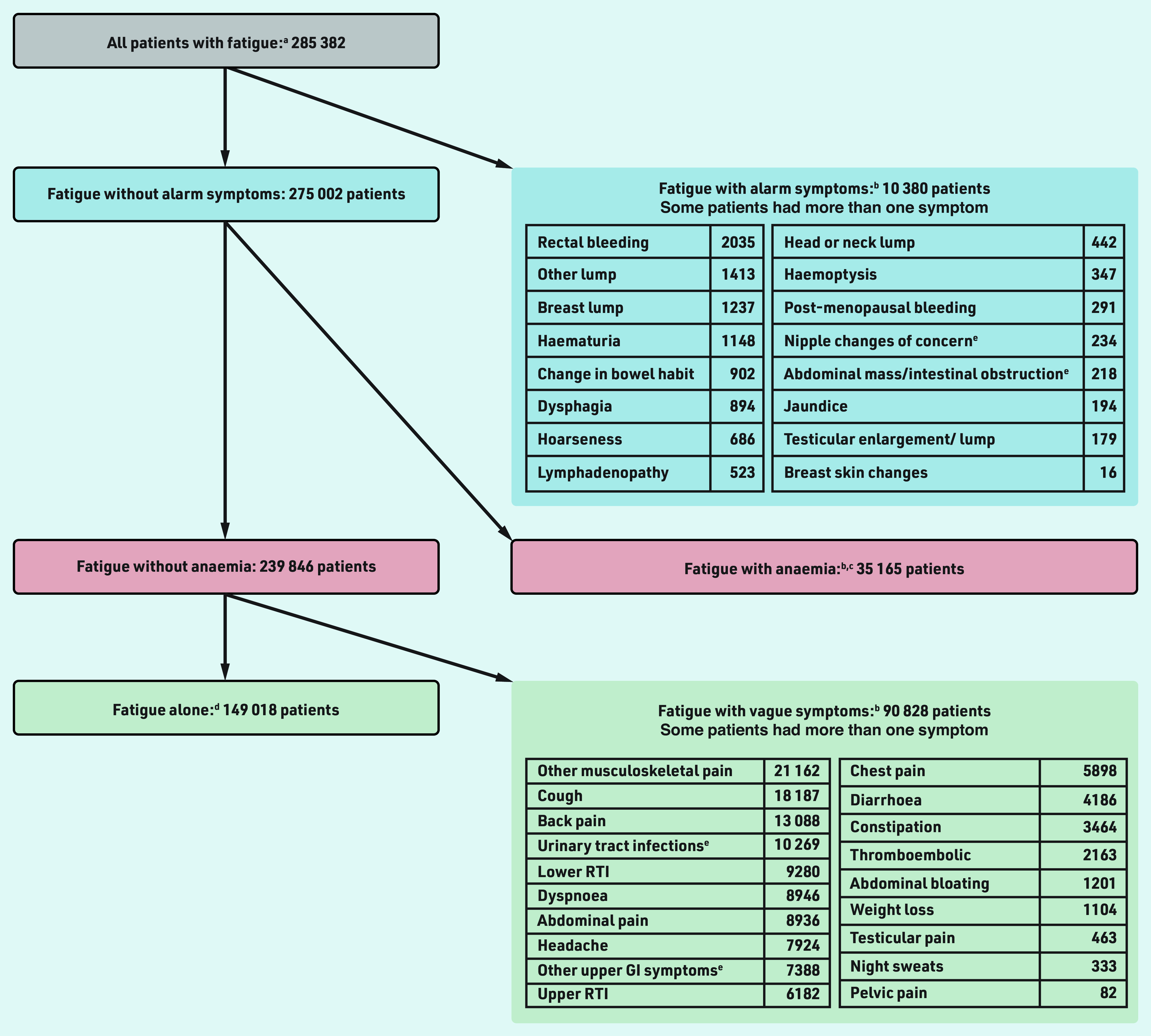
*Study cohorts. ^a^Patients with ≥1 eligible fatigue record in CPRD between 1 January 2007 and 2 April 2015. Fatigue records were eligible if occurring after the practice was ‘up to standard’ and the patient was registered to the practice for >1 year, the patient was ≥30 years, before the practice’s last collection date, the patient left the practice, turned 100 years, or died. There also had to be no fatigue record or cancer diagnosis within the previous year. ^b^Symptoms/tests were ‘co-occurring’ meaning they were recorded 3 months before to 1 month after the patient’s first eligible fatigue record. Co-occurring symptoms/tests were eligible if occurring after the practice was ‘up to standard’ and the patient was registered to the practice, and before the practice’s last collection date, the patient left the practice, died, or was diagnosed with cancer. ^c^Patients had ≥1 valid low haemoglobin measurement meeting the above eligibility criteria, and the measurement was considered valid (that is, within a biologically plausible range). ^d^Without any of the studied alarm or vague symptoms, or anaemia. ^e^Abdominal mass/intestinal obstruction also includes rectal mass. Nipple changes of concern also include nipple discharge or retraction. Urinary tract infection also includes cystitis, dysuria, urgency, painful urination, urine smell. Other upper GI symptoms include dyspepsia, nausea, vomiting, haematemesis, loss of appetite. CPRD = Clinical Practice Research Datalink. GI = gastrointestinal. RTI = respiratory tract infection.*

In addition to fatigue, 64 ‘potential’ cancer symptoms were identified from those listed in the 2011 and 2015 NICE recommendations for suspected cancer^11^^,^^21^ and additional sources.^28^^–^30 Additional symptoms of interest did not need to be established by prior literature as fatigue related. Read code lists were available for 35 of the identified symptoms, which were therefore included in the study.^2^^,^^31^^–^40 Of the additional 35 symptoms included in the study, 16 were categorised as ‘alarm’, defined as those with the NICE NG12 (2015) recommendations for urgent two-week wait referral or investigation for suspected cancer.^11^^,^^21^ The remaining 19 symptoms were categorised as ‘vague’ (Figure 1). Supplementary Table S1 lists the sources used to define each symptom, including fatigue, with all Read codes available at https://github.com/rmjlrwh/Fatigue. Of the 28 potential cancer symptoms that were not profiled because of unavailable Read code lists, 12 were categorised as ‘alarm’ and 16 as ‘vague’. These are listed in Supplementary Table S2.

People who presented with fatigue without an alarm symptom but with anaemia (defined as a low haemoglobin test result, using published methods,^2^^,^^41^ see Supplementary Box S1) were analysed separately, as anaemia in older patients would usually prompt urgent referral under the NICE NG12 (2015) guidelines.^11^^,^^21^

### Cohort identification

First, a cohort of patients aged 30–99 years presenting to primary care with new-onset fatigue between 2007 and 2015, and no cancer diagnosis in the previous year were identified in CPRD (Figure 1). The steps taken to define this cohort are detailed in a previous publication.^22^

Patients with fatigue with a ‘co-occurring’ alarm symptom (occurring between 3 months before and 1 month after the first fatigue presentation) were excluded from subsequent age-specific analysis. Patients with fatigue and no alarm symptoms were characterised for presence of ‘co-occurring’ anaemia.

Finally, for patients with fatigue and no alarm symptoms or anaemia, subcohorts of patients with fatigue and each co-occurring vague symptom were identified. These cohorts were not mutually exclusive, that is, the same patient could be in more than one cohort if they had more than one symptom combined with fatigue.

A time window of 3 months before to 1 month after the first fatigue presentation was chosen to define ‘co-occurrence’, because patients’ diagnostic episodes could span multiple visits to the doctor over a short period of time, and doctors may not record all presenting symptoms during each consultation. Records of additional symptoms or anaemia were considered ‘eligible’ if meeting the criteria detailed in Figure 1.

### Follow-up and outcomes

Follow-up began with the patient’s first eligible record of fatigue during the study period and ended either at 9 months or the first cancer diagnosis, if earlier. As NCRAS data were used to define the outcome patients could remain in the study even after they left their GP practice or their practice exited CPRD. Patients could not subsequently re-enter the study with another fatigue record.

The main outcome was diagnosis of cancer recorded in NCRAS data within 9 months following the first fatigue presentation. Nine months was chosen following a previous publication’s findings that excess cancer risk is concentrated in this period.^22^ Cancers included any malignant neoplasms excluding non-melanoma skin cancer (ICD-10 codes C00-C99 excl. C45). Benign brain tumours were not included.^22^

### Statistical analyses

Cancer risk for patients with and without alarm symptoms or anaemia were calculated, and, in the cohort of patients without, risk for each ‘fatigue–co-occurring vague symptom’ subcohort were calculated. Analysis was stratified by sex but not age band because of sample size constraints. Instead, Poisson regression models were fitted, with cancer diagnosis as the outcome and age modelled as a continuous exposure variable using restricted cubic splines, and cancer risk at selected ages modelled. Robust standard errors were used to account for possible overdispersion.

Residuals were plotted to ascertain model fit in each co-occurring symptom group. Potential interactions were observed between age and weight loss, and age and abdominal bloating (females only), but the addition of interaction terms did not improve model fit, so these were not included. As a result of small sample sizes, pelvic pain and night sweats were not included in age-specific analyses.

To contextualise modelled age-specific cancer risk estimates, 9-month cancer risk in the general population is also shown (derived using incident cancer registration statistics for England in 2011^42^ and corresponding mid-year population estimates).^43^

As a result of data availability, these were for 5-year age bands and all ages ≥85 years were grouped together. Data management and analysis were conducted in MySQL Workbench v6.1 and Stata v17, respectively.

All relevant code is available online at https://github.com/rmjlrwh/Fatigue. The Strengthening the Reporting of Observational studies in Epidemiology (STROBE) guidelines for cohort studies were used.^44^

### Sensitivity analyses

A sensitivity analysis examined the impact on cancer risk estimates of varying the time window used to define symptom co-occurrence before the first fatigue presentation, up to 12 months pre- presentation.

## RESULTS

### Cohort inclusions and exclusions

In total, 285 382 patients had ≥1 ‘eligible’ record of fatigue in primary care within the patient’s inclusion period, without a cancer diagnosis or fatigue record in the previous year (Figure 1). There were 10 380 (3.6%) patients with fatigue who had a co-occurring alarm symptom 3 months before to 1 month after their first eligible fatigue record. Of the remaining patients, 35 165 (12.8%) had anaemia.

Overall, 239 846 (84%) patients with fatigue did not have any alarm symptoms or anaemia. Of these (*n* = 239 846), 90 828 (38%) had ≥1 co-occurring vague symptoms. Approximately half (52%, 149 018/285 382) of all patients with fatigue had fatigue alone, that is, all other potential (alarm and vague) cancer symptoms studied were absent.

### Frequency of co-occurring vague symptoms

**Figure 2.**
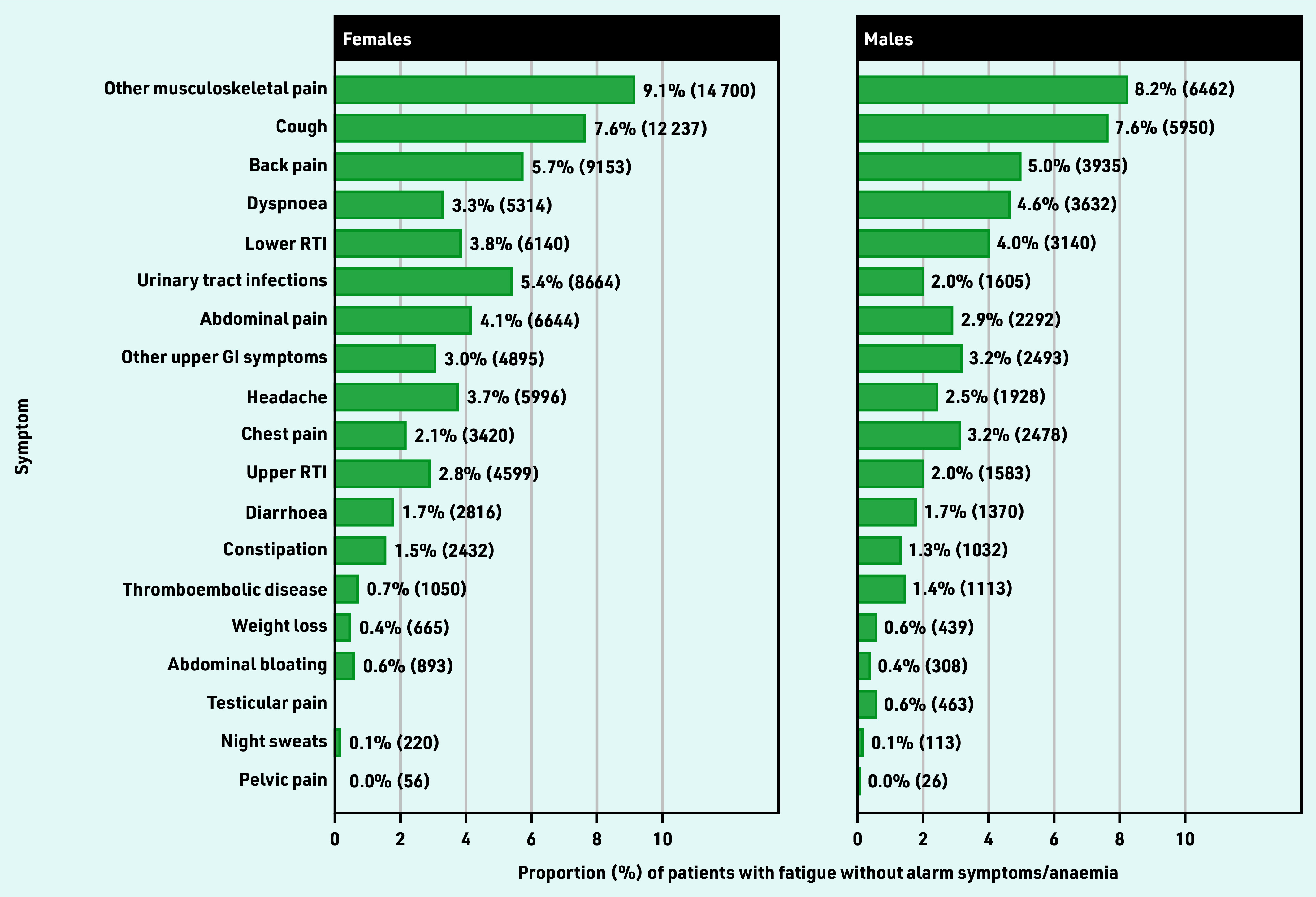
***Patients with each co-occurring vague symptom, as a proportion of patients with fatigue and no alarm symptoms or anaemia (%). Co-occurring symptoms were those recorded 3 months before to 1 month after the first fatigue presentation. Total*** n ***was 161 375 for females and 78 471 for males. These cohorts were not mutually exclusive; 12% of patients had >1 of these vague symptoms. Urinary tract infections also include cystitis, dysuria, urgency, painful urination, urine smell. Other upper GI symptom includes dyspepsia, nausea, vomiting, haematemesis, loss of appetite. GI = gastrointestinal. RTI = respiratory tract infections.***

Among patients with no alarm symptoms or anaemia (*n* = 239 846), the five most common vague symptom combinations were fatigue–musculoskeletal pain, fatigue–cough, fatigue–back pain, fatigue–dyspnoea, and fatigue–lower respiratory tract infections (Figure 2). Of patients with fatigue and no alarm symptoms or anaemia (*n* = 239 846), 26% (n = 62 732) had only one additional type of vague symptom in combination with fatigue, and 12% (n = 28 096) had ≥2 (for example, fatigue with abdominal pain and cough) (Supplementary Table S3). The cohort size and median age (interquartile range [IQR]) of the studied vague symptom combinations with fatigue are presented in Table 1.

**Table 1.** Age characteristics of patients with fatigue, with each co-occurring symptom, for all patients with fatigue, patients with fatigue, without alarm symptoms, and patients with fatigue, without alarm symptoms or anaemia^a^

**Characteristic**	**Females**		**Males**	

	**Total *n***	**Age, years, median (IQR)**	**Total *n***	**Age, years, median (IQR)**
**All patients with fatigue**	**192 614**	**52 (41 69)**	**92 768**	**58 (46 71)**
With alarm symptoms	6916	53 (42–69)	3464	63 (48–76)

**Patients with fatigue, without alarm symptoms**	**185 698**	**52 (41 69)**	**89 304**	**58 (46 71)**
With anaemia	24 323	59 (43 78)	10 833	76 (66 83)

**Patients with fatigue, without alarm symptoms or anaemia**	**161 375**	**52 (41 67)**	**78 471**	**56 (44 68)**
With vague symptoms	62 300	56 (43 71)	28 528	59 (47 72)
Without vague symptoms (that is, fatigue only)	99 075	50 (40 64)	49 943	54 (43 65)
*Pairwise combinations of fatigue with each vague symptom*				
Abdominal pain	6644	51 (40 66)	2292	57 (45 69)
Abdominal bloating	893	53 (42 69)	308	59 (46 70)
Dyspnoea	5314	68 (55 78)	3632	68 (57 77)
Night sweats	220	53 (44 65)	113	57 (49 67)
Weight loss	665	65 (48 79)	439	63 (50 76)
Constipation	2432	65 (46 80)	1032	71 (60 80)
Cough	12 237	58 (45 71)	5950	61 (48 72)
Diarrhoea	2816	60 (44 76)	1370	59 (46 72)
Pelvic pain	56	42 (38 55)	26	55 (43 65)
Other upper GI symptoms	4895	59 (45 72)	2493	58 (45 70)
Urinary tract infections	8664	60 (44 76)	1605	70 (55 80)
Other musculoskeletal pain	14 700	57 (45 71)	6462	59 (48 70)
Chest pain	3420	58 (46 72)	2478	59 (48 71)
Testicular pain			463	52 (42 65)
Headache	5996	47 (38 59)	1928	51 (41 62)
Back pain	9153	53 (42 68)	3935	56 (45 68)
Upper RTI	4599	50 (40 63)	1583	55 (43 66)
Lower RTI	6140	61 (48 75)	3140	64 (51 76)
Thromboembolic disease	1050	74 (62 83)	1113	69 (59 77)

a *Co-occurring symptoms were those recorded 3 months before to 1 month after the first fatigue presentation. Urinary tract infections also include cystitis, dysuria, urgency, painful urination, urine smell. Other upper GI symptom includes dyspepsia, nausea, vomiting, haematemesis, loss of appetite. GI = gastrointestinal. IQR = interquartile range. RTI = respiratory tract infections.*

### Cancer risk in patients with and without alarm symptoms

For all patients with fatigue (all ages combined), observed cancer risk within 9 months after first fatigue presentation was 2.2% (95% confidence interval [CI] = 2.1 to 2.3) in males and 1.1% (95% CI = 1.0 to 1.1) in females.

Risk was higher for those with alarm symptoms than those without (Supplementary Figure S1, Supplementary Tables S4 and S5).

### Cancer risk in patients with and without anaemia

**Figure 3.**
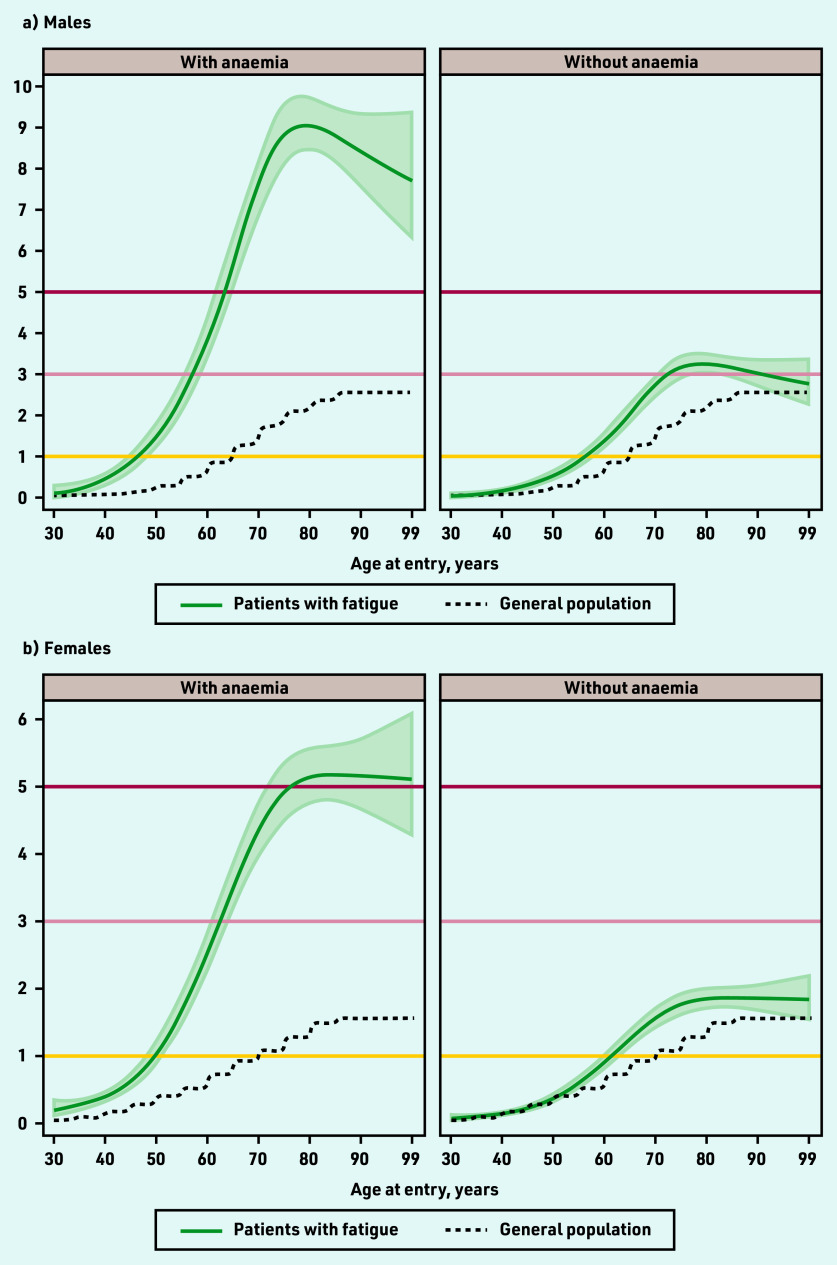
*Modelled 9-month cancer risk (%) in patients with fatigue and no alarm symptoms, for each year of age (30–99 years), by presence of anaemia. a) Males; b) females. Risk for non-linear continuous age modelled using restricted cubic splines. Includes observed 9-month cancer risk (%) for the general population in England in 2011, by 5-year age band. Available population estimates grouped all males/ females aged ≥85 years.*

For patients with fatigue and no alarm symptoms, observed cancer risk was higher for those with anaemia than those without (Supplementary Figure 1, Supplementary Tables S4 and S5). Modelled age-specific risk for patients with anaemia exceeded 3% in males from 57 years (3.1%, 95% CI = 2.7 to 3.6) and females from 62 years (3.0%, 95% CI = 2.7 to 3.4%), and 8% in males from 71 years (8.1%, 95% CI = 7.4 to 8.9%) (Figure 3, Supplementary Table S6).

### Cancer risk in patients with each vague symptom

For patients with fatigue and no alarm symptom or anaemia, observed cancer risk for all ages combined was higher for people who presented with fatigue who had ≥1 co-occurring vague symptom compared with those without. Cancer risk was higher for patients with ≥2 different additional vague symptoms in combination with fatigue (males: 2.5%, 95% CI = 2.2 to 2.9%; females: 1.3%, 95% CI = 1.2 to 1.5) compared with those with only one additional vague symptom (males: 1.5%, 95% CI = 1.4 to 1.7; females: 0.8%, 95% CI = 0.8 to 0.9) (Supplementary Table S3). For 16 out of 17 fatigue–co-occurring symptom combinations studied in females, and 15 out of 18 in males, at least a third of cancers diagnosed were for cancer sites other than the three most common in that symptom cohort (data not shown; analysis excluded symptom combinations with no cancer cases).

**Figure 4.**
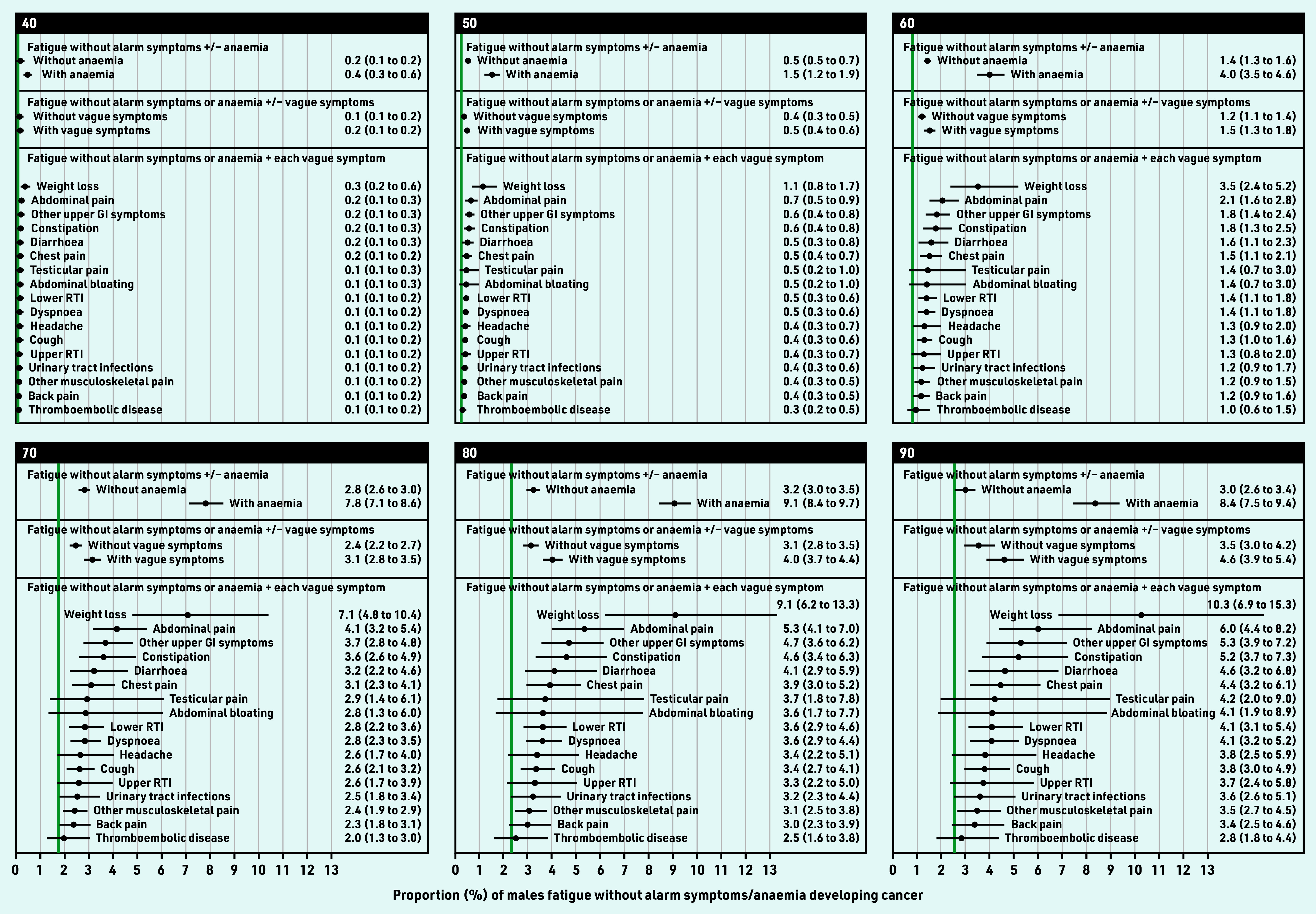
*Modelled 9-month cancer risk (%, with 95% CI) in males with fatigue and no alarm symptoms, by presence of anaemia or each co-occurring vague symptom, for selected ages. Green line = observed 9-month cancer risk (%) for the general population in England in 2011, by 5-year age band. Available population estimates grouped all males aged ≥85 years. Urinary tract infections also include cystitis, dysuria, urgency, painful urination, urine smell. Other upper GI symptom includes dyspepsia, nausea, vomiting, haematemesis, loss of appetite. CI = confidence interval. GI = gastrointestinal. RTI = respiratory tract infections.*

**Figure 5.**
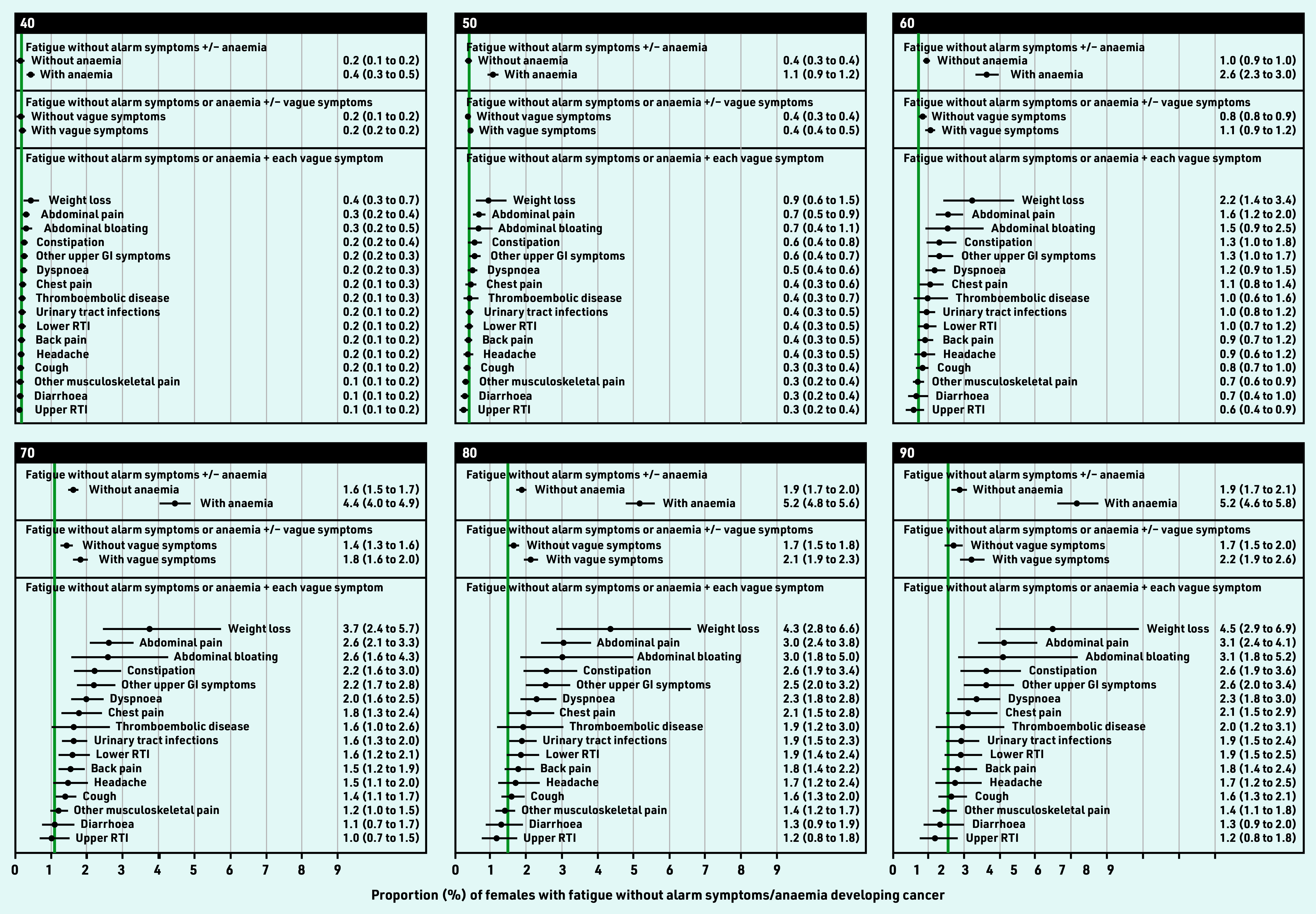
*Modelled 9-month cancer risk (%, with 95% CI) in females with fatigue and no alarm symptoms, by presence of anaemia or each co-occurring vague symptom, for selected ages. Green line = observed 9-month cancer risk (%) for the general population in England in 2011, by 5-year age band. Available population estimates grouped all females aged ≥85 years. Urinary tract infections also include cystitis, dysuria, urgency, painful urination, urine smell. Other upper GI symptom includes dyspepsia, nausea, vomiting, haematemesis, loss of appetite. CI = confidence interval. GI = gastrointestinal. RTI = respiratory tract infections.*

Overall, for all ages combined, observed cancer risk was highest for weight loss, constipation, dyspnoea, abdominal pain (males), or abdominal bloating (females) (Supplementary Figure S1, Supplementary Table S4). Age-specific modelled cancer risk increased with age for each vague symptom (Figure 4 and Figure 5, Supplementary Figure S2). Adjusting for age, cancer risk was higher for fatigue in combination with any vague symptom compared with fatigue without co-occurring vague symptoms (Supplementary Table S5). These combinations included four specific symptoms in males, and six in females: fatigue–weight loss, fatigue–abdominal pain, fatigue–constipation, fatigue–other upper gastrointestinal (GI) symptoms, fatigue–abdominal bloating (females), or fatigue–dyspnoea (females).

In males, the age at which risk exceeded 3% was 59 years (3.2%, 95% CI = 2.2 to 4.7) for fatigue–weight loss; 65 years (3.1%, 95% CI = 2.4 to 4.1) for fatigue–abdominal pain; 67 years (3.1%, 95% CI = 2.2 to 4.2) for fatigue–constipation; and 67 years (3.1%, 95% CI = 2.4 to 4.1) for fatigue–other upper GI symptoms.

In females, risk exceeded 3% from 65 years (3.1%, 95% CI = 2.0 to 4.7) for fatigue–weight loss; and 79 years (3.0%, 95% CI = 2.4 to 3.8%) for fatigue–abdominal pain; and 80 years for fatigue–abdominal bloating (3.0%, 95% CI = 1.8 to 5.0%) (Table 2, Supplementary Figure S2, Supplementary Table S7).

**Table 2.** Age (years) at which modelled 9-month cancer risk (%) exceeded 2%, 3%, and 6% in patients with fatigue without co-occurring alarm symptoms/anaemia, by presence of each co-occurring vague symptom^a^

**Characteristic**	**Males, age in years**			**Females, age in years**		

	**Risk >2%**	**Risk >3%**	**Risk >6%**	**Risk >2%**	**Risk >3%**	**Risk >6%**
**Patients with fatigue, without alarm symptoms or anaemia**						
With vague symptoms	63	70	—	75	—	—
Without vague symptoms	67	78	—	—	—	—

**Pairwise combinations of fatigue with each vague symptom**						
Abdominal bloating	65	72	—	64	80	—
Abdominal pain	60	65	90	64	79	—
Back pain	67	80	—	—	—	—
Chest pain	64	70	—	77	—	—
Constipation	62	67	—	68	—	—
Cough	66	75	—	—	—	—
Diarrhoea	63	69	—	—	—	—
Other upper GI symptoms	61	67	—	68	—	—
Dyspnoea	65	72	—	71	—	—
Headache	66	74	—	—	—	—
Lower RTI	65	72	—	—	—	—
Other musculoskeletal pain	67	79	—	—	—	—
Testicular pain	64	71	—	—	—	—
Thromboembolic disease	71	95	—	99	—	—
Upper RTI	66	75	—	—	—	—
Urinary tract infections	66	77	—	—	—	—
Weight loss	55	59	67	59	65	—

a *Urinary tract infections also include cystitis, dysuria, urgency, painful urination, urine smell. Other upper GI symptom includes dyspepsia, nausea, vomiting, haematemesis, loss of appetite. GI = gastrointestinal. RTI = respiratory tract infections.*

### Sensitivity analyses

In the main analysis, co-occurring symptoms were identified if recorded 3 months before to 1 month after the patient’s first fatigue presentation. In sensitivity analysis, broadening the look-back time window to 12 months before fatigue presentation resulted in substantial increases in the proportions of people who presented with fatigue who had both accompanying alarm symptoms and accompanying vague symptoms (Supplementary Table S8). This resulted in slightly lower risk of cancer, consistently across all symptom combinations examined (Supplementary Table S9).

## DISCUSSION

### Summary

In patients presenting to primary care with fatigue without alarm symptoms or anaemia, the frequency of 19 co-occurring vague symptoms were characterised. Age-adjusted cancer risk was higher for those with any vague symptom studied compared with fatigue without co-occurring vague symptoms, including four specific symptoms in males and six in females. Cancer risk exceeded 3% in older males with fatigue and any vague symptom, reaching this threshold earliest for fatigue–weight loss (59 years), fatigue–abdominal pain (65 years), fatigue–constipation (67 years), and fatigue–other upper GI symptoms (67 years). For females, risk exceeded 3% only in older females with fatigue–weight loss (65 years), fatigue– abdominal pain (79 years), and fatigue–abdominal bloating (80 years).

### Strengths and limitations

This study has a number of strengths. It uses high-quality electronic health records from CPRD, which are broadly representative of the age, sex, and ethnicity distribution of the UK population.^45^ Linkage to population-level cancer registration (NCRAS) data offered ‘gold standard’ ascertainment of cancer diagnoses.^46^ Unlike most similar studies,^18^^,^^19^^,^^23^^,^^24^^,^^47^^–^60 the 9-month follow-up for cancer was guided by previous evidence of the duration of increased cancer risk following first fatigue presentation.^22^ This study also demonstrated for the first time that cancer risk estimates would be lower if using longer look-back periods before the first fatigue presentation for including co-occurring symptoms.

There are several limitations to this study. The study population is limited to patients who presented to primary care with fatigue and in whom their doctors deemed the symptom severe enough to be coded in their records^61^ and does not represent the broader population of patients who experience fatigue in the community.^62^ Therefore, comparisons with the general population are intended only to contextualise risk.^22^ GPs are most likely to code a symptom if they deem it to be serious.^61^ Therefore, fatigue may also have been present (although not recorded) in consultations when more serious (for example alarm/red-flag) symptoms were present. Such patients would not have been included in the current study population.

Fatigue was examined in combination with other potential cancer symptoms, where Read code lists were available for those symptoms. It is possible that a small minority of patients included in the cohort of patients with fatigue and no alarm symptom had one of 12 alarm symptoms for which Read code lists were unavailable (Supplementary Table S2); however, the symptoms that were not included are likely to occur rarely in practice. Future research could examine a wider range of alarm and vague symptoms using more recently available Read code lists^63^ or lists developed in other coding systems.^64^

Age- and symptom-specific risk estimates were produced through the use of modelling. However, the number of patients with some co-occurring symptoms (for example, abdominal bloating in males) and at some ages — especially age ≥90 years — was small, resulting in imprecision of some age–symptom-specific risk estimates.

Although not possible in this study because of sample size limitations, further stratification of exposures would be informative, for example, by morbidity status, the nature of co-occurring symptoms (for example, chronic or recent onset), or by multiple combinations of symptoms (for example, fatigue in combination with abdominal pain and abdominal bloating). Furthermore, the risk of all cancers combined was examined, whereas NICE guidelines are usually based on the risk of a specific cancer site.

### Comparison with existing literature

To the authors’ knowledge, this is the first study to characterise symptom co-occurrence in people who presented with fatigue, and to estimate cancer risk in patients with fatigue and a wide range of vague symptoms. Together with other evidence, the findings establish abdominal pain, weight loss, and fatigue as vague symptoms that confer a substantial risk of cancer often exceeding normative risk thresholds, particularly in combination.^22^^–^25^,^^53^^,^^59^ Nevertheless, it should be noted that the mere presence of additional vague symptoms is a marker of elevated risk, particularly if two or more are present.

In addition, older males with fatigue–constipation or fatigue–other upper GI symptoms (which included dyspepsia, nausea, vomiting, haematemesis, loss of appetite) and older females with fatigue– abdominal bloating were also at elevated (>3%) risk of cancer. This is concordant with prior literature examining some of these abdominal symptoms either alone^53^^,^^59^ or in combination with weight loss^23^ or abdominal pain.^25^

### Implications for research and practice

This study illustrates the feasibility of producing cancer risk estimates for groups of patients with symptoms that co-occur with a vague symptom, such as fatigue. The detailed examination of cancer risk in patients presenting to primary care with new-onset fatigue in the absence of alarm symptoms for cancer can guide the management of a sizeable population of patients for whom diagnostic management is particularly challenging. On average, in older males, the presence of other vague symptoms, additional to fatigue, increases the risk of undiagnosed cancer to levels exceeding specialist referral thresholds (>3%) recommended by NICE in the UK. In older females, risk for certain combinations of vague symptoms (fatigue–weight loss, fatigue–abdominal pain, and fatigue–abdominal bloating) exceed these thresholds.

This study examined patients with fatigue and other vague cancer symptoms. By their nature, vague symptoms are likely associated with a moderately raised risk for many different cancer sites. In this study, even the top three sites diagnosed following a fatigue–vague symptom combination typically excluded at least a third of cancers diagnosed. This varied mix of cancers also meant the ranking of cancer sites was not precise, as there were often several sites forming similar proportions of cancers diagnosed. In a different sample these could be expected to be ordered differently, hence it could not be said with certainty whether the risk of any one cancer site was higher than others. Future research aiming to identify the most common sites in patients with combinations of vague symptoms should use larger sample sizes and consider incorporating further risk stratifiers (for example, results of common blood tests) that could further differentiate between the most likely cancer sites.

The current study also found that, in patients with fatigue and no alarm symptoms, cancer risk exceeds 3% in patients with anaemia, rising to over 8% in older males. Although anaemia type (for example, by iron deficiency status) was not characterised in this study, the findings indicate that low haemoglobin alongside fatigue confers a relatively high risk of cancer, which is supported by previous research into anaemia.^65^ Although fatigue can be directly attributable to anaemia, it is important that the risk of underlying cancer in these patients is also investigated, particularly in older patients. Although existing NICE guidelines recommend appropriate investigation of anaemia, this alarm feature is not always appropriately investigated.^66^

In conclusion, fatigue is not usually recorded in primary care in combination with an alarm symptom for cancer. The age- and sex-specific risks reported in this study can guide clinical decisions about referrals for specialist investigations for cancer, depending on the presence or absence of other vague symptoms presenting alongside fatigue.

## Data

The data used in this study were accessed through the CPRD, and is subject to protocol approval by an Independent Scientific Advisory Committee, and therefore cannot be directly shared. Data management and analysis code, and Read code lists, are available online at https://github.com/rmjlrwh/Fatigue.
